# Participation in everyday life for young people with chronic pain in Saudi Arabia: “you feel lacking in life and you feel that time is flying by”

**DOI:** 10.3389/fresc.2023.1099345

**Published:** 2023-05-19

**Authors:** Fatimah Alsaggaf, Imelda Coyne

**Affiliations:** ^1^ Nursing College, King Abdulaziz University, Jeddah, Saudi Arabia; ^2^ School of Nursing and Midwifery, Trinity College Dublin, Dublin, Ireland

**Keywords:** chronic conditions, young people, pain experience, quality of life, chronic pain, pain management strategies

## Abstract

**Introduction:**

Chronic pain is a common health problem that can have a significant impact on children and young people's daily life. Although research on pediatric chronic pain has been a priority globally, little is known about young people's experience of chronic pain in Saudi Arabia. Thus, this article reports on young people's experience of chronic pain and the impact on their lives in Saudi Arabia which forms part of a larger study.

**Methods:**

Multiple case study design following Yin's (2018) approach was used. Purposeful and theoretical sampling were used to recruit young people aged 12 to 18 who had experienced chronic pain for at least three months, their parents, and their school personnel. The young people and their parents were recruited from a tertiary hospital located on the western side of Saudi Arabia while school personnel were recruited from the schools that young people attended. Data were collected through in-depth semi-structured face-to-face (*n* = 15) and telephone interviews (*n* = 25) from 40 participants (10 young people, 10 parents, and 20 school personnel). Interviews were recorded, transcribed verbatim, and translated from Arabic to English. Data were analyzed following two phases: (1) constant comparative analysis; and (2) cross-case analysis based on the work of Charmaz (2014) and Yin (2018) respectively.

**Findings:**

Young people's experiences of chronic pain were categorized into three themes: (1) experiencing chronic pain; (2) impact of pain on quality of life; and (3) everyday strategies to manage chronic pain. All young people reported that their pain was caused by a chronic condition, where the most prevalent pains were musculoskeletal/joint pain, abdominal pain, and headache/migraine. Most young people had encountered challenges with misdiagnosis or delayed diagnosis as to the cause of their chronic pain. They described how their chronic pain interfered with their physical, psychological, and social functioning. They primarily managed their pain with medications and through self-care techniques. The findings also indicated that young people's generally positive attitude to their pain reflected their beliefs in Allah's power and the belief that such suffering should be borne according to their Islamic culture.

**Conclusion:**

Chronic pain is a significant health phenomenon that tends to restrict the participation of young people in everyday life. However young people used a range of strategies to normalize the pain so that they could continue with their everyday activities like their peers.

## Introduction

Chronic pain is a worldwide health problem (1, 2) and a common experience among children and young people (1, 3–6). The prevalence of chronic pain in children and young people is high (4, 6), ranging from 14% to 76% (7–10). Gobina et al. (1) compared the prevalence of chronic pain internationally among young people (*n* = 214,283) and reported that 11.3% of the targeted population experienced headache, 7.7% backache, and 4.6% abdominal pain. Pain is defined as “an unpleasant sensory and emotional experience associated with, or resembling that associated with, actual or potential tissue damage” (11) and chronic pain is described as pain that persists for three months or more (12–14). Chronic pain can be caused by an earlier injury or ongoing health condition (15, 16) and it may also be an idiopathic illness or a symptom of a non-progressive disease, or it can be the chronic condition of pain itself (15). It often leads to high utilization of health care services by children and young people with pain (4, 17–19). A recent systematic review reported frequent visits to the emergency department, for children and young people experiencing episodes of pain crises and exacerbations (17). Similarly, international studies showed increased visits to emergency rooms (18, 19), outpatient clinics, and increased inpatient hospitalizations for young people with chronic pain (18). Furthermore, children and young people often experience untreated chronic pain and encounter delays in being seen and receiving chronic pain care (20, 21).

Research indicates that chronic pain also has a widespread negative impact on children and young people's quality of life (2, 13, 22, 23). It affects physical functioning leading to functional disability, interrupting performance of daily physical activities (2, 5, 6, 22, 24, 25), disturbing sleep patterns (25, 26), and diminishing psychological well-being (5, 22, 24, 25) leading to high levels of emotional distress, anxiety, depression, and mood disturbances (2, 14, 26, 27). In addition, young people's chronic pain can influence social functioning, affecting relationships with family and friends and contributing to social isolation (2, 5, 22, 24, 25). Moreover, school functionings of children and young people, including school attendance, academic performance and competence, physical functioning, and social functioning, appeared to be negatively impacted by chronic pain (28).

According to the World Health Organization, young people with chronic pain should be treated as having a serious health issue that requires active management (29) using a multidisciplinary approach (1). A UK study found that multidisciplinary treatment followed by pharmacological interventions were effective strategies in managing young people's (*n* = 95) pain (30). Similarly, a Swedish study found that 74% of children and young people (*n* = 69) used analgesics to manage their pain (31). Previous studies have also reported the use of non-pharmacological interventions for children and young people with chronic pain. In Andersson et al.'s study, 58% of children and young people (*n* = 69) used a variety of complementary and alternative medicine to alleviate pain (31), for instance, nonvitamin, nonmineral dietary supplements (32), thermotherapy and massage (33), hydrotherapy (33, 34), and healthy diet (34). Relaxation (33, 35, 36) and cognitive behavioral therapy (37) were also reported to be effective psychosocial treatments for children and young people with pain. Young people with chronic pain may also benefit from exercising (33, 34) to reduce their pain. A further self-pain-management strategy that has been used by young people lately are online resources (38), such as Youtube (39), WebMAP (40), and iCanCope (41, 42) chronic pain applications.

Although a large body of research on pediatric chronic pain exists globally, there is a deficit of research on young people's experiences of chronic pain in Saudi Arabia. Thus, this paper reports on the experience of chronic pain, its impact on participation in everyday life, and strategies to manage chronic pain from the perspectives of young people and their parents in Saudi Arabia.

## Materials and methods

This paper adhered to the consolidated criteria for reporting qualitative research (COREQ) guidelines reported by Tong, Sainsbury, and Craig (43). The researchers adopted a social constructivist stance where reality and knowledge are socially shaped by how humans perceive the world and how they interact within it (44). Yin's approach (45) was selected to conduct the multiple case study because it offered a good structure for how a case study research should be conducted, analyzed, and reported. Arguably, Yin's post-positivist stance does not align with the constructivist stance chosen to investigate the research aim, which was to explore the reality of chronic pain phenomenon among young people in Saudi Arabia, its impacts on school functioning, and its management in school settings. However, Yin's approach (45) allowed the researchers to understand “how” young people experienced chronic pain, “how” and “why” chronic pain impacted young people's school functioning, “how” their pain was managed in school settings and “why” such strategies were used. Carrying out a multiple case study as proposed by Yin (45) allowed the researchers to collect triadic perspectives (young people, parents, and school personnel) to better understand the phenomenon of chronic pain. This created the opportunity to address the gap in the body of knowledge being researched.

The multiple case study approach entailed collecting data through semi-structured interviews (*n* = 40) to capture multiple perspectives and to obtain in-depth data regarding the chronic pain phenomena from more than one case “unit of analysis” (young people with chronic pain, their parents, and school personnel). The boundaries for the cases were determined by the study's settings (a clinical setting and schools of participating young people) and contexts (young people, their parents, and school personnel). The interview guide contained open-ended questions structured around the study objectives, so participants could elaborate on the topics during the interviews. In addition, the multiple case study facilitated comparison between cases and identification of similar and contradictory findings. This study was part of a larger study exploring the chronic pain experience of young people, the impact of pain on school functioning, and pain management interventions in school settings.

### Sampling, recruitment and participants

Purposive and theoretical sampling were used to recruit young people aged between 12 and 18 who met the criteria of experiencing chronic pain for at least three months, their parents, and their school personnel. The initial number of young people who met the above criteria was 25; however, 15 young people and their parents refused to take part mainly due to ethical considerations. For example, some mothers were reluctant to take part because the interviews were being recorded. They were reluctant to have their voices on tape because of cultural norms about modesty (i.e., propriety in dress speech or conduct) in the conservative Islamic religion.

Purposive sampling was the first strategy for selecting participants since it is the most useful type of sampling in case studies (46), especially when the researcher wants to get a deeper understanding of a phenomenon (47) from informative, unique cases or a particular population, or when there is a need for an in-depth investigation about certain cases (46). An initial sample of seven young people, consisting of five females and two males, with an equal distribution of the severity of their chronic pain, were recruited using purposeful sampling. In addition, their parents, including six mothers and one father, and fourteen members of their school personnel, were recruited in this purposive sampling phase.

Then, theoretical sampling was used as it is the consecutive process which takes place when the initial data collection and analysis have been determined, and thus the emergent codes and categories inform the theoretical sampling, and data saturation is reached (48, 49). Theoretical sampling was chosen to elaborate further on developing codes and categories and test their relationships by recruiting more participants. This involved analysing the data and generating a sampling frame specifying the participants' characteristics (e.g., pain location, age, or gender), which assisted in informing who to sample next and what probing questions should be asked in the interview. Therefore, further information was obtained from the newly recruited participants until data saturation was achieved. Theoretical sampling was accomplished by focusing on a heterogeneous sample that included fathers of young people with chronic pain, more male young people, young people with abdominal and/or musculoskeletal pain, younger age groups (12–15 years old), and those who were enrolled in middle schools. Thus, at this sampling stage, three additional young people—two males and one female—were sampled and interviewed; two of them had abdominal pain while the third had musculoskeletal pain. Two mothers, one father, and six members of the school personnel were also interviewed.

Young people with chronic pain and their parents were recruited from one of the largest tertiary care hospitals in western Saudi Arabia, Jeddah, while school personnel were recruited from the schools of participating young people, which were located in either Jeddah or Makkah cities in Saudi Arabia. The gatekeepers for recruiting young people and their parents were senior nurses and consultants from the female and male adult inpatient wards, pediatric inpatient wards, and outpatient clinics. The school managers of the schools were contacted after written permission was sought from young people and their parents, and they were requested to identify assistant principals or counselors and schoolteachers, who had cared for the participating young people or who had experienced supporting students with chronic pain. The gatekeepers distributed an information letter to eligible participants that included information about the study and the researcher's contact details to help young people, parents and school personnel understand the research aims and whether to decide to participate. Participants then made contact with the researcher who explained the study again and reiterated the confidentiality and privacy issues. In addition, in order to conduct research involving young people, the researcher first obtained permission and written consent from the parent; this was then supplemented with the young person's assent. The study was conducted in the period between May 2020 to November 2020.

### Ethical approval

The Faculty of Health Sciences Ethics Committee at Trinity College of Dublin, Dublin, Ireland, and the biomedical ethics committee at the largest tertiary care hospital in the western region of Saudi Arabia, in Jeddah, reviewed and approved the study. Additional approval was obtained from Ministry of Education in Jeddah and Makkah, Saudi Arabia.

### Data collection

Participants were invited to face-to-face, telephone, or online interviews using Microsoft Team since the data collection was carried out during the COVID-19 pandemic, assisting to overcome issues like time constraints and restricted public health measures that were raised in response to the pandemic. Most participants preferred telephone interviews as they were cost- and time-effective, especially for those living in remote cities, and because they were a safer choice during the COVID-19 pandemic. Moreover, although the use of online interviews for qualitative research has significantly increased, using Microsoft Team was a new software in Saudi Arabia, where most participants felt uncomfortable to consider for data collection.

The first author, FA, a female PhD candidate and nurse lecturer with expertise caring for pediatric clients, conducted all the interviews with young people, their parents, and school personnel sensitively and facilitated open and safe communication with the participants. Fifteen face-to-face interviews and 25 telephone interviews were conducted, and each interview lasted approximately 35–85 min. All the interviews were conducted individually to avoid any potential influence due to the presence of a third party (e.g., a parent) and to ensure open communication. Face-to-face interviews were conducted in settings of the participants' preference, namely an examination room in the hospital clinics for young people and their parents and in the student counselor's room in school settings for interviews with school personnel. All interviews started with an open-ended question “Tell me about your pain”, “Tell me about your young person's pain” or “Tell me about your experience of working with a young person experiencing chronic pain”.

Interviews were audio-recorded, transcribed verbatim, and translated from Arabic to English for analysis. Since each script may have multiple viable meanings when translated into another language, the principal researcher is a native Arabic speaker who can understand and communicate a variety of cultural and social expressions. Furthermore, the researcher chose an excerpt from a transcript and invited a colleague who is proficient in both Arabic and English to cross-check the translation. The colleague then assured the accuracy of translated scripts. Participants' records, including audio records and transcripts, were pseudonymized, where real names were removed, and pseudonyms were given for young people participants. For example, “Adam” was assigned for one of the young people, “Adam's mother” for his mother, and “Adam's schoolteacher” for his schoolteacher.

### Data analysis

The first author, FA, reviewed the interview transcripts, while the second author, IC, constantly supervised and reviewed all stages of the data analysis. A combination of Charmaz's (50) constant comparative analysis, and Yin's (45) cross case synthesis analysis, was followed to analyze the data. As explained earlier, Yin's approach (45) provides a clear framework for how to analyze multiple case studies through cross case synthesis. In addition, Yin's approach (45) allows the flexibility to combine different methods of analysis and select the best suited for analyzing case study research. Thus, Charmaz's (50) constant comparative analysis was found to be the most suitable and thorough method for analyzing the large amount of data obtained by using line-by-line coding.

Constant comparative analysis (50) was conducted in three stages: (1) open coding where a list of initial codes was generated by carrying out comparison within each interview; (2) focused coding where similarities and differences within each case and across cases were identified, and categories developed; and (3) comparison across cases to generate themes and comprise relevant categories. Each of the three stages of data analysis discussed above was electronically sorted using the data analysis program Nvivo 12, which assisted in grouping related data into thematic sets. Cross-case synthesis was subsequently used through examining each case study separately and then aggregating the findings in accordance with a common framework of categories or themes that emerged from the individual cases (45). For the purpose of this paper, the data related to impacts of chronic pain on school functioning and pain management interventions in school settings were not included; therefore, this paper reports only on young people's experiences of chronic pain.

## Findings

Young people who participated in this study included five males and five females, and their ages ranged from 12 to 16 years. Both mothers and fathers were invited to participate; and eight mothers and two fathers participated. Parents had between two and eight children, with most having three children. Sample demographics are provided in Tables 1, 2.

**Table 1:** Demographic characteristics of the young people who participated.

Young people names (pseudonyms used)	Gender	Age	Chronic Condition Diagnosis	Pain Onset	Pain Location	Pain Intensity
Adam	Male	13	Sickle cell anemia	Toddler	One site	Severe
Hala	Female	16	Sickle cell anemia	Toddler	Multiple sites	Severe
Lara	Female	12	Celiac disease	Preschool	Multiple sites	Mild to severe
Areej	Female	16	Systematic lupus erythematous and migraine	Primary school	Multiple sites	Mild to severe
Ehab	Male	16	Osteogenesis imperfecta	Birth	Multiple sites	Mild-severe
Jumana	Female	16	Chronic sinusitis and headache	Primary school	Multiple sites	Severe
Zahed	Male	16	Muscular dystrophy	Primary school	Multiple sites	No pain to mild
Sedra	Female	15	Juvenile rheumatoid arthritis (multiple sites)	Primary school	Multiple sites	Mild to severe
Akram	Male	14	Ulcerative colitis disease	Middle school	Multiple sites	Mild-severe
Majed	Male	14	Crohn's disease	Primary school	One site	Mild-severe

**Table 2:** Demographic characteristics of the parents who participated.

Parents names (pseudonyms used)	Gender	Age	No. of Children	Socio-economic Status	Level of Education
Adam's mother	Mother	40	6	Married/Housewife	Bachelor
Hala's mother	Mother	46	3	Married/Housewife	High school
Lara's mother	Mother	49	6	Married/Housewife	Bachelor
Areej's mother	Mother	47	3	Married/Housewife	Middle school
Ehab's mother	Mother	59	8	Married/Housewife	Middle school
Juman's mother	Mother	40	3	Divorced/Employed	Bachelor
Zahed's father	Father	46	3	Married/Employed	Middle school
Sedra's father	Father	50	2	Married/Employed	Bachelor
Akram's mother	Mother	38	4	Married/Housewife	High school
Majed's mother	Mother	43	4	Married/Housewife	High school

Young people's experiences of chronic pain were categorized into three themes: (1) Experiencing chronic pain: “Like a big rock on me… I cannot bear it”: (2) Impact of pain on quality of life: “Pain ruins everything”: and, (3) Everyday strategies to manage chronic pain: “I don’t let the pain disrupt my day”.

### Theme 1. Experiencing chronic pain: “Like a big rock on me… I cannot bear it ”

Young people and parents explained the experience of chronic pain. Two categories were developed: (1) describing pain which details the characteristics of young people's pain (see Table 1); and (2) seeking help from healthcare professionals in which the experiences of misdiagnosis and delayed diagnosis were discussed.

#### Describing pain

For all young people, chronic pain principally emerged from their chronic conditions. The age of chronic pain onset ranged from birth to 12 years, while most young people experienced pain worsen as they grew older. Some young people felt pain either at one site, such as their back, which was the most common site, followed by the abdomen and head. Others felt it at multiple sites. Pain episodes were noted to occur daily, especially before receiving a medical diagnosis for the chronic conditions, whereas when the pain was controlled, it became intermittent and each pain episode would last for a few seconds to a week. Young people explained their pain intensity as ranging from mild to severe pain. Seven young people had faced mild pain while six had experienced severe pain and three were still struggling with severe pain.

Young people used a variety of evocative terms to describe their pain ranging from someone who was “hitting” (Adam), “squeezing” (Hala and Akram), “pressing” (Hala, Lara, Jumana, and Akram), “pressuring” (Jumana), or “breaking” (Areej) them. Whilst others described their pain as “a big rock” (Hala) or “a heavy weight” (Jumana), indicating that it was a significant burden on them. “As it were a big rock on me… I cannot bear it. I feel something holding me by force… I mean, pain holds me everywhere. I feel someone is squeezing me” (Hala).

Almost all young people reported that that their chronic pain was triggered by physical activities (e.g., walking) and/or environmental conditions (e.g., cold and/or hot weather). For instance, a young person spoke about how the cold weather would exacerbate her pain. “[When] there are lots of cold weather … the pain comes … and it raises so much… it will be strong” (Akram).

Other triggers for pain included pain and chronic condition management, diet, injuries, insufficient sleep, stress or sadness, and studying. Also, physiological changes in puberty intensified the pain of two young females (Hala and Areej) with pain. “When the menstrual period comes to Areej, the first day, she is in a state… she cries and is in pain” (Areej's mother).

#### Seeking help from healthcare professionals

All young people with pain sought professional help for their pain, especially during the onset or the peak of pain. Health care utilization was used mainly to determine the cause of their pain and to obtain help for their pain. Almost all young people encountered difficulty understanding the cause of their chronic pain, and they had experienced misdiagnosis and delayed diagnosis for their chronic conditions during the onset of pain. A mother whose son had received a final diagnosis of Crohn's disease stated that, during the onset of the chronic condition, a pediatric physician thought that her son's pain was associated with a psychological issue. “At first … I took Majed to a children doctor… She [did] for him [blood] tests and told me, “There is nothing, he is good. [But] his hemoglobin is a little bit low… [And] she said, “Maybe it is psychological and on Allah's willing, he will get better” (Majed's mother).

Experiencing misdiagnosis and delayed diagnosis of chronic conditions appeared to impact significantly on almost all young people, leading to repeated diagnostic evaluation procedures, delay in medical diagnostic and treatment procedures, and consequently delay in managing pain. One mother described the challenges of repetitive invasive and non-invasive procedures before finally receiving a conclusive medical diagnosis for her son. “The whole period [10 days], he was hospitalized, and every day they were… drawing blood samples… doing [blood] analysis… and they do not know what the reason is… what problem he has” (Adam's mother).

The delay in medical diagnostic (e.g., x-ray and endoscopy) and medical treatment (e.g., surgery) procedures were also reported by some young people (Ehab, Jumana, Akram, and Majed) and their parents. For instance, one mother described long periods of her child struggling with chronic pain due to the cancellation of the endoscopy appointment and the physician's failure to see the necessity of conducting such a diagnostic procedure.

Another mother reported a constant battle with physicians to obtain surgery for her daughter, and that, in turn, had exacerbated her pain. *“*So, when I took Jumana [to the doctor], he told me, “Leave her two years after puberty and [then] come to see [us]. This is adenoid… She [must] reach 18 years of age.” I mean, see [now] her [health condition] symptoms are increasing” (Jumana's mother).

### Theme 2. Impact of pain on quality of life: “Pain ruins everything”

Pain impacted the quality of life for young people in many ways. They and their parents described how pain affected the physical, psychological, and social parts of young people's lives. It was apparent that experiencing pain influenced young people's lives. Some young people (Adam and Hala) who had sickle cell anemia reported significant life disruption due to chronic pain. *“*You feel lacking in life, and you feel that time is flying by … Pain ruins everything” (Hala).

#### Impact on physical functioning

The interference of chronic pain with physical functioning was a common struggle faced by most young people, leading them to be immobile and to remain sitting most of the time. Three of the young people (Ehab, Zahed, and Sedra) had emphasized the experience of using a physical aid during the peak of pain or being dependent on a wheelchair. “[While] we [the family] were walking in a mall, Sedra's walking was disrupted…[So,] we had to look for a wheelchair [to] push her … because she was saying, “I cannot walk” … [due to] joint pain” (Sedra's father).

The reduction in young people's physical functioning presented challenges with daily activities, such as helping with home chores. Performing five daily prayers involving standing, raising hands, kneeling, and sitting was also a challenge, especially for the young person whose pain resulted from juvenile rheumatoid arthritis. “She cannot pray; she cannot prostrate. And it became difficult for her to kneel” (Sedra's father).

The physical impact of pain also affected how young people performed enjoyable activities. Four young people (Adam, Hala, Sedra, and Akram) described how their chronic pain inhibited them from performing well in activities they enjoy, such as playing soccer, before the onset of pain or pain progression. “But with the pain during this period, I became cold when playing [soccer]. I no longer play as [I used to]” (Adam).

The energy level was another issue described by some young people and their parents. Pain left young people feeling exhausted, fatigued, lacking energy, and needing to rest, hindering them from living normally. “I did not have power. I was not getting up” (Majed).

Sleep disturbances, including sleep deprivation, were an issue for most young people. Five mothers (Adam's, Hala's, Ehab's, Jumana's, and Akram's mothers) reported that pain prevented their young person from having a good sleep due to repeatedly waking up at night. *“*He wakes up from his best sleep complaining” (Akram's mother).

Eating problems were an additional issue reported by several young people and their parents. Loss of appetite and stomach upsets (e.g., nausea and vomiting) were experienced by five young people (Adam, Hala, Areej, Akram, and Majed) primarily when their pain flared up. *“*He cannot eat anything” (Adam's mother).

#### Impact on psychological functioning

Young people's psychological functioning was adversely affected by their pain. Young people and their parents reported several psychological symptoms such as anxiety and changes in mood, changes in memory and concentration, as well as altered body image and physical appearance.

Anxiety primarily resulted from worries of sudden pain episodes. Changes in mood related to pain were also reported by young people. Three mothers (Areej's, Akram's and Majed's mothers) reported their young persons' loss of interest in engaging in enjoyable activities, such as cooking and playing. In addition, some young people (Adam, Hala, Areej, and Sedra) and parents, commonly reported feelings of anger and agitation, stubbornness, nervousness, unexplained crying, and mood fluctuations. “I get bothered so easily. I get nervy … I used to be patient before” (Areej).

Some young people also experienced negative thoughts about the pain. The pain was seen as a “crisis” (Hala), “problem” (Adam), “very critical” (Adam and Akram), “unsafe” (Adam, Jumana, Sedra, and Majed), “very torturing” (Adam), and “spirit ruining” (Hala).

Five young people (Adam, Hala, Areej, Jumana, and Majed) experienced difficulty with memorization and concentration. They and their parents explained how their memory and concentration had worsened since the onset of pain and how they had struggled to stay focused. For example, one young person spoke about the effort she had to make to maintain concentration when someone spoke to her. “No matter what I tried, whatever was important [about] this person's words, I could not concentrate” (Sedra).

Young people also spoke about various bodily changes and altered body image resulting from pain and pain treatments. Two young people (Adam and Sedra) experienced changes in physical experience, such as hair loss, limping gait, and functional impairment (with the need to use a wheelchair). All these bodily changes affected how young people perceived their bodies. For example, a young person talked about how ashamed she felt when she perceived people's stigmatization for her limping gait. “When I was limping … I hated people's stares… I didn’t want them to stare at me. I was feeling embarrassed… that I was young, [but] I can’t walk” (Sedra).

#### Impact on social functioning

Young people with chronic pain experienced impaired social functioning, mainly social isolation. All young people and their parents noted social isolation. Pain was the principal associated factor of being socially withdrawn among most young people. For example, a young person stated that she would isolate herself when her pain flared up so she would not let her pain affect people close to her. “When I have severe headache, and I am not active with anything that happens in the day … [so] instead of being cranky and [irritable to those] who surround me… I isolate myself” (Jumana).

Two young people (Akram and Majed) and their parents noted communication difficulties due to young people's pain intensity. A mother stated her son was not having conversations with her or his siblings during the peak of pain. “Even when I used to talk to him, he stared at me like that. I tell him, “Hey, do you see me?” So, he tells me, “Yes, but I do not have the power to speak”… Even there was no talking between him and his siblings” (Majed's mother).

### Theme 3. Everyday strategies to manage chronic pain: “I don’t let the pain disrupt my day”

Despite the difficulties young people were facing due to living with chronic pain, they were still attempting to manage the chronic pain through medications and self-care management strategies.

#### Taking medications for pain

Most young people and their parents mentioned the appropriate use of medications to manage pain. One young person described how he was continuously ensuring sufficient supply of pain medication at home to relieve his pain. “At home, I keep lots of extra [pain relief] patches” (Adam).

Adhering to pain medications was also described by young people and parents. One mother stated that she always kept a painkiller in her daughter's school backpack as the daughter's physician recommended it to avoid pain flaring up. “I always put a painkiller in her backpack…[If] she feels pain, she takes it. Because the doctors said, “Once the pain comes to her, let her take it till she comes to the hospital. So, the pain will not increase” (Hala's mother).

One young person additionally described her day and night routines of taking medications before going to bed or leaving home to ease pain and go about her daily life. “I take pills before I sleep to benefit me [while] I am sleeping… [and] before I go out of the house” (Jumana).

#### Self-care management strategies

In addition to pain medications, young people and parents found a broad range of strategies to manage young people's pain. Young people often used more than one strategy, although not all strategies were successful. The self-care management strategies included alternative therapies, cognitive strategies, bearing pain, physical activity, spiritual strategies, family support, and avoidance of pain triggers. Most young people used alternative therapies such as herbal remedies, applying massages to the pain area, using hot or cold compressions, drinking water, and eating healthy food.

All young people used cognitive strategies to overcome their pain. These included positive thinking, relaxation, and distraction. Positive thinking about pain was described as “accepting the pain” (Areej and Jumana), “being used to pain” (Jumana), “living life” (Hala and Lara), “do not let it disrupt you” (Jumana), “it bothers, but it is okay” (Areej), “it is not dangerous” (Ehab), “a source of strength” (Hala), and “teaches you a lesson” (Hala). For example, one young person explained that although the pain negatively impacted her life, she would bear it to continue with her daily life. “I don’t let the pain disrupt my day… I don’t let the pain disrupt my studying” (Jumana).

Relaxation was another cognitive strategy that most young people described. Almost all young people found that lying down and sleeping were helpful to make their pain subside. “As soon as I lay down or sleep … the second day, I come back [feeling] better” (Ehab).

Distraction was also one of the cognitive strategies, though this was less commonly used by young people. Three young people (Adam, Lara, and Majed) and their parents noted how they used enjoyable activities (e.g., watching on the phone, talking, joking with friends, or drawing) to distract their thinking about pain. “I do not spend time thinking about pain, as it would hurt me more. For example, I draw … so [as] to occupy myself” (Lara).

Young people and their parents described how most young people were trying to bear their pain, despite how well they could control it. One young person who was using painkillers for managing his pain explained: “When it came [to me] the pain, I did not put on the [pain relief] patches. I said, “In the name of Allah, I want to try.” … I stay trying to control the pain, but I could not” (Adam).

Three young people (Areej, Jumana, and Majed) reported exercising, such as walking, as a self-care strategy to manage pain. One young person revealed that participation in physical activities eased his pain. “I walk a little bit, so the pain goes away” (Majed).

Spirituality also played a role in helping four young people (Adam, Hala, Akram, and Majed) to manage their pain. Most parents explained how their spirituality of being Muslims helped them to cope as a parent having a young person struggling with chronic pain. For example, a mother discussed her belief in Allah's plans and his blessings as well as her acceptance to Allah's commands and destinies for having a son with chronic pain. “Allah writes what it has good[ness] … Glory be to Allah, if our Allah loves a believer, … “He afflicted him” … So, thank Allah. I accept the decree of Allah and his destiny… [And] I ask Allah … to have a mercy on Ehab's weakness” (Ehab's mother).

Parents' believing in Allah seemed to influence positively on their young people's coping with pain. One mother described how she used to encourage her son to remember that having a chronic pain is an Allah's command and to be hopeful that Allah would ease his pains. The young person accordingly mentioned his belief in Allah's plan, praying and hoping that Allah would alleviate his pain.

*I tell him, “Pain is a Allah's command. Thank Allah. [And] on Allah's will, you will get better…And this pain, on Allah's will, will be eased” (Adam's mother)*

*Maybe this pain is Allah's goodwill; you do not know… Sometimes I say, “Allah, [I hope] it does not come to me this pain” (Adam)*

All young people experienced family support which had a significant role in easing the pain experience and overcoming challenges associated with pain. For instance, one young person stated the help she received from her sister and mother in doing homework and explaining school course materials when missing school. “Sometimes I ask my sister [to do homework] … I [also] let my mother explains to me” (Lara).

Avoidance of pain triggers, such as avoidance of physical activities, hot or cold weather, and adherence to a restricted diet, was a common strategy used by young people. Avoidance of physical activities was the most common strategy noted by young people with chronic pain. They reported that they were sitting and resting when needed and reducing movement to avoid pain exacerbation. “I can go upstairs… I get tired a little bit, but then I stop a bit for five minutes and then I continue going upstairs” (Hala).

Avoidance of environmental changes, hot or cold weather, was mentioned by the mother of a young person whose pain was caused by sickle cell anemia. She stated the avoidance of mountainous places where the weather is usually cold.

Adherence to a restricted diet was a common self-care strategy among young people (Lara, Akram, and Majed) with abdominal pain. One mother described the diet regime of her daughter since receiving the diagnosis of celiac disease and the significance of acknowledging the food ingredients to avoid pain episodes. “Whatever I buy, I must know which companies are certified gluten-free… eight years… and … we have been following the diet” (Lara's mother).

## Discussion

These findings illustrate how young people experienced their chronic pain and how pain negatively impacted several aspects of their daily lives. However, young people were constantly trying to manage their pain to improve their overall well-being and normalize their participation in everyday life. Young people described their chronic condition-related pain as having a mild to severe intensity and affecting one or more bodily regions. These findings are comparable to those of a recent international survey (*n* = 214,283) that Gobina et al. undertook to find out how prevalent chronic pain is among young people (1). According to the study, young people frequently experience both single- and multi-site chronic pain (1). Young people and their parents also identified individualized pain triggers in the current study, which were caused by environmental, physical, pharmaceutical, emotional, and physiological factors. These factors were also reported in a previous study conducted with young people with chronic pain (*n* = 100) (51). They reported that a variety of biopsychosocial factors triggered their pain with a high frequency associated with physical trauma, medical condition or disability, and surgery or other medical procedures (51).

Seeking help from healthcare professionals was consistent with previous studies' findings that young people seek medical advice to determine the cause of pain and find appropriate pain treatments (4, 17–19, 52). Young people and their parents experienced misdiagnosis and delayed diagnosis despite the importance of understanding the cause of chronic pain which has been reported elsewhere (53–55). The concept of misdiagnosis and delayed diagnosis were associated with multiple diagnostic procedures, protracted wait times for medical diagnostic and treatment procedures, and a lack of knowledge about options for managing chronic pain. These findings accord with a Finnish study by Sulkanen et al., who found that abdominal pain among young people with inflammatory bowel disease (e.g., ulcerative colitis and Crohn's disease) predicted a delayed diagnosis for more than six months (53). Similarly, another study discovered that most young people (*n* = 17; 13–20 years) with systemic juvenile idiopathic arthritis had misdiagnosis in which their pain symptoms were misinterpreted as “growing pains” or psychological issues or misdiagnosed with other serious chronic conditions (54). The current study's findings suggest that the concept of misdiagnosis and delayed diagnosis were associated with healthcare professionals' dismissing the pain experienced by young people. This resulted in a delay in diagnosing and treating chronic pain and more suffering and potentially worse health outcomes for young people experiencing pain. Pain dismissal was seen as resulting from the challenge to diagnose and treat chronic pain as it frequently manifests with few or no objective physiological symptoms, and assessment almost entirely depends on client reports (55, 56). These findings highlight how crucial it is for healthcare professionals to better understand, define, and treat pediatric pain. It was also suggested that receiving a medical diagnosis or validation would assist young people living with pain to understand and manage their health conditions (57, 58).

The findings revealed that chronic pain impacted on young people's quality of life, especially their participation in daily life, including their physical, psychological, and social functioning. Similarly, other studies have found that chronic pain negatively impacts the health-related quality of life of young people (59–62). In the current study, chronic pain affected young people's sleep quality and energy level, leaving them immobile and fatigued, experiencing sleep disturbances, and limiting their participation in daily and enjoyable activities, such as home chores, praying, and playing. Similarly, several studies reported that lack of sleep (59, 63, 64) and feeling fatigued (63, 65) due to chronic pain were associated with restricting participation in physical activities for young people (63, 66, 67). Psychological dysfunction was also reported by young people in this study as affecting their participation in daily life activities. Most young people reported experiencing anxiety and changes in mood, which were also identified in other studies conducted with young people complaining of chronic pain (60, 68, 69). A study by Gil et al. in USA indicated that young people (*n* = 37; 13–17 years old) with sickle cell disease who were found to have a high level of stress and a negative mood were also experiencing an increase in daily pain, which resulted in a decrease in participation in daily social and home activities (69). Social functioning, including social withdrawal, was additionally noted in this study to affect young people's social activities. The severity of chronic pain was highly related to young people's impairment of social functioning which is similar to earlier studies in which young people with chronic pain experienced social isolation and peer bullying (70), and negative friendship quality (71).

These study's findings may therefore be used to provide and promote more effective treatments and education to improve the physical, psychological, and social well-being of young people with chronic pain. The findings also emphasized the role of employing cognitive behavioral therapy as an effective strategy to manage young people's pain, pain related distress, and disability.

Young people and their parents reported how young people used pain management strategies to push themselves to engage in daily activities for as long as possible. Young people and their parents reported adherence to prescribed pain medications to control pain episodes. This finding aligns with the studies of Fouladbakhsh et al. (72) and Harbaugh et al. (73), reporting medication use to treat pain among young people. A significant finding in this study was the varied self-treatment strategies used by young people. One common strategy was the use of alternative therapies, mainly herbal remedies, which may be due to positive attitudes in Saudi Arabia's culture and people towards complementary and alternative medicine (74). In one study, Saudi young people (*n* = 736) aged between 15 and 19 years old reported using complementary and alternative medicine to treat health conditions, such as abdominal pain (75). Cognitive strategies, such as positive thinking about pain, were another coping mechanism among young people. The notion of positive thinking and/or optimism and their roles in reducing pain and pain catastrophizing, and improving quality of life, were also reported in several studies (76–78).

In this study, all young people reported that family support helped them to cope with pain. It appears that the families of young people were contributing positively toward coping with chronic pain (79–82). Another coping strategy that some young people and their parents used was the influence of spirituality and religion in managing pain. Although there is limited research on the role of spiritualty and religions in managing pediatric chronic pain, in Baetz and Bowen's study with participants (*n* = 37,000) aged 15 years old and older with chronic pain in Canada, they found that spiritual and religious individuals used positive coping mechanisms more than non-religious people (83). The results of the current study suggest that living in an Islamic culture where individuals believe in Allah's power and plan and hope and trust in Allah was a significant factor in coping with pain. The Islamic religion seemed to provide spiritual and emotional support for those young people, and provided them with hope, optimism, and encouragement to fight against the restrictions of their conditions and chronic pain. Such results are in line with prior research indicating that young people with chronic conditions who trusted in Allah, strengthened their relationship with him through prayers or religious activities, and believed that he was merciful and loving felt less alone, were relieved and comforted, and had a more optimistic attitude about their health (84). The current study's findings imply that providing young people with chronic pain with the proper support and information from healthcare professionals may help to increase their use of healthy coping mechanisms. The findings may also assist healthcare professionals in developing appropriate culturally sensitive pain management strategies that can facilitate normalization strategies and foster resilience among young people with chronic pain. Further research on the influence of Islamic culture on pediatric chronic pain management is recommended.

### Limitations

There are a number of limitations to this study that should be considered. The study included young people who had secondary chronic pain brought on by a variety of chronic conditions, which may limit the applicability of the findings to young people experiencing primary (idiopathic) chronic pain. Young people and their parents were recruited from a large tertiary care hospital in the western region of Saudi Arabia, which may explain the limitation of identifying eligible adolescents with primary (idiopathic) chronic pain. For future research, recruiting participants from school communities might assist in identifying students with primary (idiopathic) chronic pain. As was previously indicated, the study was carried out during the upsurge of COVID-19 pandemic, which meant that in-person interviews were discouraged. As a result, of the strict public health measures, participants were offered the option of having face-to-face online interviews using Microsoft Teams (the only platform ethically approved for conducting online interviews) or telephone interviews. It could be argued that online face-to-face interviews would have allowed the researcher to decipher non-verbal clues through observation of body language, facial expression, and eye contact, which may have enhanced comprehension of what was being said. However, all of the participants, who declared difficulty with in-person and online interviews, chose telephone interviews. This might be influenced by the possibility of not needing to dress modestly in accordance with the conservative Islamic Saudi culture, especially for female participants. In addition, the workload of having distance learning, where participants (young people, parents, and school personnel) were required to sit for long hours to meet the need of the school work had affected the participants' preference for telephone interviews. Moreover, the flexibility in re-scheduling the time and date of telephone interviews was perceived to improve participation rates. Transportation restriction issued by Saudi government in response to the spread of the COVID-19 pandemic and digital health services that were implemented to help people receive medical care and prescriptions remotely from their homes may have influenced participants' perspective and experiences of pain.

### Implications

This paper presents the first publication that explores young people's chronic pain experience and participation in everyday life in Saudi Arabia. Recognizing the impacts of pain on young people's lives and its management may raise awareness of the significant demand for a multidisciplinary approach when working with young people living with chronic pain. The multidisciplinary approach where biopsychosocial pain development is considered would help young people with chronic pain to participate to their desired extent in their everyday lives. In order to assist with the management of chronic pain, the multidisciplinary approach should also be aware of sociocultural variables, such as the importance of religious beliefs and spirituality in young people's lives. Further research regarding chronic pain among young people should include national mixed method studies of individual support required by young people experiencing chronic pain in Saudi Arabia.

## Conclusion

Since chronic pain is a significant health phenomenon, this multiple case study provided an in-depth exploration of chronic pain experience among young people who live in Saudi Arabia. The findings indicate that the parents and healthcare professionals working with young people with chronic pain are central to the experience of chronic pain of young people. It appears that parents' and healthcare professionals' responses to young people's pain could hinder or support young people's managing their pain and participating in everyday life. Receiving empathetic support from both parents and healthcare professionals assisted young people to strive for normalcy, be resilient, and continue with their everyday activities despite experiencing chronic pain.

## Data Availability

The original contributions presented in the study are included in the article/Supplementary Material, further inquiries can be directed to the corresponding author/s.
